# Mass Spectrometry-Based Metabolomics in Formalin-Fixed Paraffin-Embedded Skin Biopsies Identifies Potential Candidate Biomarkers for Leprosy Progression Across the Ridley–Jopling Clinical Spectrum

**DOI:** 10.3390/microorganisms14071567

**Published:** 2026-07-17

**Authors:** Noriel Viana Pereira, Bruno de Carvalho Dornelas, Willian Vargas Tenório da Costa, João Paulo Sanches Zana, Edmundo Nunes dos Santos Araújo, Felipe dos Anjos Rodrigues Campos, Deiriene Rodrigues de Oliveira Campos, Tiara da Costa Silva, Hebreia Oliveira Almeida de Souza, Mário Machado Martins, Luiz Ricardo Goulart Filho, Isabela Maria Bernardes Goulart

**Affiliations:** 1 Graduate Program in Health Sciences, School of Medicine, Federal University of Uberlândia, Uberlândia 38400-902, MG, Brazil; noriel@ufu.br (N.V.P.); imbgoulart@ufu.br (I.M.B.G.); 2 Pathology Unit, Hospital das Clínicas, Federal University of Uberlândia, Brazilian Hospital Services Company (HC-UFU/HU Brasil), Uberlândia 38405-302, MG, Brazil; dcampos@ufu.br; 3 Department of Anatomy and Surgery, Hospital das Clínicas, Ribeirão Preto Medical School, University of São Paulo, Ribeirão Preto 14048-900, SP, Brazil; willian19vtc@gmail.com; 4 School of Medicine, Federal University of Uberlândia, Uberlândia 38400-902, MG, Brazil; jpszana@ufu.br (J.P.S.Z.); filipin_anjos@ufu.br (F.d.A.R.C.); 5 Pathology Unit, Hospital das Clínicas, Federal University of Minas Gerais, Brazilian Hospital Services Company (HC-UFMG/HU Brasil), Belo Horizonte 30130-100, MG, Brazil; edmundo.araujo@ebserh.gov.br; 6 Institute of Exact, Natural Sciences and Education, Federal University of Triângulo Mineiro, Uberaba 38064-200, MG, Brazil; tiara.silva@uftm.edu.br; 7 Institute of Biotechnology, Federal University of Uberlândia, Uberlândia 38405-320, MG, Brazil; hebreia@ufu.br (H.O.A.d.S.); mario.martins@ufu.br (M.M.M.); lrgoulart@ufu.br (L.R.G.F.); 8 National Reference Center for Leprosy and Sanitary Dermatology (CREDESH), HC-UFU/HU Brasil, Uberlândia 38405-302, MG, Brazil

**Keywords:** leprosy, *Mycobacterium leprae*, metabolomics, liquid chromatography–mass spectrometry, biopsy, paraffin embedding, real-time PCR, enzyme-linked immunosorbent assay

## Abstract

Leprosy presents a broad clinical–immunological spectrum, whose heterogeneity challenges early diagnosis and disease stratification. Metabolomic approaches have emerged as promising tools for identifying potential biomarkers associated with the disease’s pathophysiology. This study aimed to investigate metabolic profiles associated with the different clinical forms of leprosy using untargeted metabolomics in formalin-fixed paraffin-embedded (FFPE) tissue samples. A retrospective cross-sectional study was conducted with 55 patients classified according to the Ridley–Jopling spectrum. Metabolites were extracted from FFPE skin biopsies and analyzed by liquid chromatography–mass spectrometry (LC-MS). From 908 metabolites initially detected, 27 were retained after frequency filtering. Six metabolites ultimately met the criteria of one-way analysis of variance (ANOVA, *p* < 0.05) and fold-change (FC ≥ 2.0) for differential expression, while N-stearoyl tryptophan was identified as an additional candidate metabolite based on its contribution to multivariate group discrimination. These included 11-hydroperoxy-H4-neuroprostane, which showed a specific association with bacterial load, and the Gly-Pro-Lys tripeptide, which correlated with markers of infection progression. Metabolomics applied to FFPE samples proved feasible for discriminating the clinical spectrum of leprosy and annotating signatures associated with immune response. This approach represents an innovative strategy for exploratory biomarker discovery using archived histopathological samples in translational research.

## 1. Introduction

Leprosy is a multisystemic infectious disease caused by *Mycobacterium leprae* and *Mycobacterium lepromatosis*, primarily affecting the skin and peripheral nerves, which results in progressive and potentially irreversible sensory and motor impairment. The disease exhibits broad clinical variability, which is directly related to the host’s immune response [1].

The Ridley–Jopling classification spectrum categorizes patients into distinct clinical forms based on histopathological and immunological criteria, encompassing tuberculoid (TT), borderline tuberculoid (BT), mid-borderline (BB), borderline lepromatous (BL), and lepromatous (LL) leprosy [2]. For therapeutic purposes, patients are grouped into paucibacillary (PB: TT, BT) and multibacillary (MB: BB, BL, LL) [3]. However, this operational classification does not always accurately reflect the inflammatory status or the therapeutic response potential of individuals. The complex interaction between innate and adaptive immune responses, modulated by genetic and environmental factors, plays a central role in the establishment of infection and disease progression [4].

Overlapping the chronic course of the disease, acute inflammatory episodes known as type 1 (T1R) and type 2 (T2R) leprosy reactions can occur in more than 50% of patients, exacerbating nerve damage and contributing to the development of physical disabilities and stigma [5].

In this context, metabolomics emerges as a promising tool for identifying potential biomarkers capable of reflecting the host’s pathophysiological status, as well as for elucidating the molecular mechanisms underlying the disease [6,7]. These metabolites, the end products of cellular activity, provide a functional representation of the organism’s physiological or pathological state, serving as phenotypic signatures [7].

Numerous studies demonstrate that metabolomic analysis can contribute to early diagnosis, therapeutic monitoring, and the development of novel treatment strategies [8,9]. This approach has been applied across various clinical conditions [10,11,12,13,14,15,16,17,18,19,20], including infectious diseases such as tuberculosis [21,22,23,24,25].

Despite these advances, a knowledge gap persists regarding the application of omics approaches in leprosy, particularly concerning the annotation of metabolites associated with the various clinical forms and the identification of potential candidate biomarkers with diagnostic and prognostic utility. The rationale for this study is based on the high clinical heterogeneity of leprosy and the untapped potential of archived formalin-fixed paraffin-embedded (FFPE) tissue samples for retrospective molecular investigations. We hypothesized that different clinical forms of leprosy across the Ridley–Jopling spectrum would exhibit distinct metabolomic signatures in skin tissue, reflecting specific host–pathogen interactions and bacterial load. Furthermore, we posited that untargeted mass spectrometry-based metabolomics could identify specific metabolites in FFPE biopsies that correlate with clinical markers of infection progression.

Accordingly, this study aimed to identify and characterize, through an untargeted metabolomics approach, differentially expressed metabolites in FFPE samples from patients representing the various clinical forms of leprosy, utilizing LC-MS.

## 2. Materials and Methods

### 2.1. Study Design and Case Selection

This is a retrospective cross-sectional study conducted at the National Reference Center for Leprosy and Sanitary Dermatology of the University Hospital at the Federal University of Uberlândia (CREDESH/HC-UFU/HU Brasil, Uberlândia, Brazil), Minas Gerais, Brazil. From a total of 674 patients newly diagnosed with leprosy between 2015 and 2020, 60 cases were selected and classified according to the Ridley–Jopling classification spectrum [2]. The time interval between the archival of the FFPE tissue blocks (2015–2020) and the subsequent metabolite extraction (performed in April 2023) ranged from approximately 3 to 8 years. All samples were stored under standardized conditions to minimize chemical degradation.

Data were collected from secondary sources obtained from the medical records of patients diagnosed and treated by leprosy specialists at CREDESH/HC-UFU. The study was approved by the Research Ethics Committee of the Federal University of Uberlândia (Opinion No. 7,255,654/2024).

Inclusion criteria consisted of patients who presented, at the time of diagnosis (prior to the initiation of treatment), with histopathological examinations including a biopsy-derived bacillary index (BI), qPCR for the detection of *Mycobacterium leprae* DNA, and anti-PGL-I IgM ELISA serology.

Exclusion criteria applied to patients with concomitant infectious comorbidities, such as tuberculosis, HIV/AIDS, and viral hepatitis, as well as those whose FFPE tissue blocks were insufficient or unavailable for further histological sectioning. Additionally, cases with concomitantly negative results for both biopsy-derived qPCR and anti-PGL-I ELISA serology were excluded.

### 2.2. Leprosy Definition and Diagnostic Criteria

**Figure 1:**
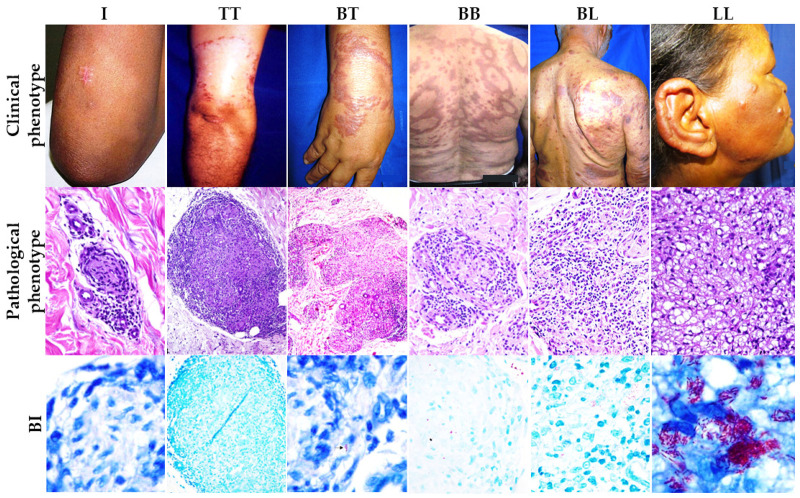
Differences in clinical phenotype, pathological phenotype, and biopsy-derived bacillary index (BI) across the clinical forms of leprosy. The six clinical forms, indeterminate (I), tuberculoid (TT), borderline tuberculoid (BT), mid-borderline (BB), borderline lepromatous (BL), and lepromatous (LL), exhibit characteristic variations in skin lesions, histopathological findings in skin biopsies, and histological bacillary load.

Leprosy can be classified into five clinical forms along the Ridley–Jopling spectrum: TT, BT, BB, BL, and LL. Additionally, an initial, non-specific presentation known as the indeterminate (I) form is recognized, although it is considered to precede the clinicopathological conditions of the classic spectrum. Patient classification was conducted based on clinical, histopathological, bacilloscopic, serological, and molecular characterization, according to the Ridley–Jopling criteria [2], including the I form (Figure 1) (Supplementary Table S1).

Following 55. participants, distributed into six groups according to clinical form: indeterminate (I, n = 8), tuberculoid (TT, n = 6), borderline tuberculoid (BT, n = 12), mid-borderline (BB, n = 13), borderline lepromatous (BL, n = 7), and lepromatous (LL, n = 9).

Four participants (A06, A07, A08, and A09—Supplementary Table S1) were excluded due to simultaneous negativity for *Mycobacterium leprae* DNA detection via biopsy qPCR and anti-PGL-I ELISA serology. Additionally, one participant (A41) was excluded due to an insufficient quantity of metabolites obtained during extraction.

### 2.3. Study Variables

Epidemiological and clinical variables were included, comprising: sex, age, clinical form according to the Ridley–Jopling classification, biopsy-derived bacillary index (BI), detection of *Mycobacterium leprae* DNA by qPCR in biopsy samples, and anti-PGL-I IgM serology.

#### 2.3.1. Biopsy-Derived Bacillary Index

The BI was determined from skin lesion samples obtained via skin biopsies. FFPE blocks were subjected to histological sectioning and stained using the Fite–Faraco method, which is the preferred technique for the identification of *Mycobacterium leprae* in tissue sections [26]. The bacillary load was expressed through the logarithmic BI, calculated according to the scale proposed by Ridley and Hilson [27].

#### 2.3.2. Anti-PGL-I IgM Serology

Serum IgM anti-PGL-I antibodies were detected via enzyme-linked immunosorbent assay (ELISA), using native PGL-I purified from the cell wall of *Mycobacterium leprae*. The reagents employed, including PGL-I and the anti-*M. leprae* monoclonal antibody, were obtained from BEI Resources (NIAID, NIH, Manassas, VA, USA; NR-19342 and NR-19370, respectively).

Anti-PGL-I antibody titers were determined at a 1:10,000 dilution, with optical density (OD) measured at 492 nm. Results were expressed as the ELISA Index (EI), calculated by the following formula: EI = ODsample/ODcutoff. Values of EI ≥ 1.0 were considered positive [28].

#### 2.3.3. Real-Time PCR for *Mycobacterium leprae* DNA Detection in Biopsies

DNA was extracted from skin biopsies previously preserved in liquid nitrogen, using the NucleoSpin^®^ Tissue kit (Macherey-Nagel^®^, Düren, Germany). Cell lysis was performed with lysis buffer and Proteinase K, followed by washing steps for the removal of impurities and subsequent DNA elution in an appropriate buffer. The quantity and quality of the obtained DNA were evaluated through quantification assays. The extracted material was then used for the amplification of the specific repetitive genomic sequence of *Mycobacterium leprae* (RLEP3), via real-time polymerase chain reaction (qPCR), utilizing the ABI 7300 system (Applied Biosystems, Foster City, CA, USA) [29,30].

#### 2.3.4. Metabolite Extraction

Following the identification of FFPE tissue blocks, the surface area of each sample was calculated in mm^2^ based on the section dimensions. The blocks had been stored in a standardized climate-controlled archive (20–25 °C, controlled humidity) at the Pathology Unit/HC-UFU since their collection (2015–2020). Subsequently, the blocks were sectioned into 10 µm thick sections.

For metabolite extraction, spectroscopic-grade methanol was added to the FFPE sections, standardizing the ratio between solvent volume and tissue area across all samples. Samples were vortex-mixed for 5 min and then centrifuged at 13,000× *g* for 15 min. The supernatant was transferred to a new microcentrifuge tube and subjected to evaporation in a vacuum concentrator for 30 min.

The dried residue was resuspended in 400 µL of methanol, vortex-mixed for 10 min, and filtered through a 0.22 µm membrane. Analyses were performed using LC-MS, utilizing an Agilent 1260 Infinity HPLC system (Agilent Technologies, Santa Clara, CA, USA) coupled with an Agilent 6520B high-resolution quadrupole time-of-flight (Q-TOF) mass spectrometer (Agilent Technologies, Santa Clara, CA, USA) equipped with an electrospray ionization (ESI) source (Agilent Technologies, Santa Clara, CA, USA).

Compound identification was based on high-resolution mass measurements, considering a mass error below 10 ppm, with comparison to database entries and literature data. Agilent MassHunter Qualitative software (version 10.0, Agilent Technologies, Santa Clara, CA, USA) was used for raw data processing, employing the Molecular Feature Extraction (MFE) tool (Agilent Technologies, Santa Clara, CA, USA) for mass spectra extraction and conversion to CEF format. Metabolite identification was performed using the METLIN database (Scripps Research Institute, La Jolla, CA, USA) integrated into MPP.

For compound alignment and filtering, Agilent Mass Profiler Professional (MPP) software version B.13.1.1 (Agilent Technologies, Santa Clara, CA, USA) was utilized. The applied criteria included a minimum abundance of 5000 counts, a minimum of two ions per compound, and the acceptance of all charge states. Alignment parameters were defined with a retention time tolerance of 0.15 min and a mass window of 15 ppm + 2 mDa. Compounds present in 100% of the samples from at least one experimental group, with a coefficient of variation (CV) below 25%, were considered for subsequent analysis. These filtering and preprocessing thresholds were selected based on established metabolomics protocols to minimize technical noise and prioritize metabolites with high analytical reliability [31,32].

### 2.4. Statistical Analysis

Metabolomic data were initially subjected to frequency filtering, considering only metabolites present in 100% of the samples from at least one group, which resulted in the selection of 27 metabolites from the 908 initially detected. The primary criterion for identifying differentially expressed metabolites during the discovery phase was one-way analysis of variance (ANOVA; *p* < 0.05) with False Discovery Rate (FDR) correction, combined with fold-change filtering (FC ≥ 2.0). Metabolomic analyses were conducted using the MetaboAnalyst 5.0 online platform, Xia Lab, McGill University, Montreal, QC, Canada (https://www.metaboanalyst.ca, accessed on 16 December 2024).

Subsequently, the individual expression intensities for the identified candidates were evaluated for normality using the Shapiro–Wilk test. Given the absence of a normal distribution (*p* < 0.1), the Kruskal–Wallis test was applied as a confirmatory inferential analysis to validate the significant differences across the six clinical forms of leprosy, adopting a significance level of 5% (*p* < 0.05). Associations between metabolite expression and markers of bacillary load and humoral response (bacillary index, anti-PGL-I ELISA index, and qPCR Ct values) were evaluated using Spearman’s correlation (*ρ*). Correlation strength was interpreted as weak (∣*ρ*∣ < 0.30), moderate (0.30–0.49), or strong (≥0.50) [33]. Multivariate analyses, including Principal Component Analysis (PCA) and Partial Least Squares-Discriminant Analysis (PLS-DA), were conducted using MetaboAnalyst 5.0 to evaluate global distribution and discriminatory capacity. Considering the Ridley–Jopling classification as an ordered clinical–immunological spectrum, a complementary trend analysis was performed to evaluate progressive variation in metabolite expression during disease evolution. For this analysis, clinical forms were numerically ordered: Indeterminate (I = 1), Tuberculoid (TT = 2), Borderline Tuberculoid (BT = 3), Mid-borderline (BB = 4), Borderline Lepromatous (BL = 5), and Lepromatous (VV = 6). The association between the clinical spectrum order and metabolite expression intensity was assessed using Spearman’s correlation (*ρ*) with a 5% significance level (*p* < 0.05).

For PCA, data matrix adequacy was initially verified using the Kaiser–Meyer–Olkin (KMO) index and Bartlett’s test of sphericity. PCA was employed as an unsupervised multivariate approach to evaluate the global distribution of the samples and identify natural grouping patterns across the clinical forms of leprosy. The variance explained by the principal components was considered adequate when the first two components accounted for between 50% and 70% of the total data variability, a range frequently described as satisfactory in metabolomics studies involving complex and heterogeneous biological systems. Values within this range indicate a consistent representation of the global data structure without excessive loss of the biological variability inherent to the analyzed samples [34,35].

Supervised multivariate analyses were conducted using the MetaboAnalyst 5.0 online platform (https://www.metaboanalyst.ca accessed on 1 July 2026). Data were subjected to sum normalization, log10 transformation, and auto-scaling to reduce technical variability and approximate normal data distribution. Subsequently, partial least squares-discriminant analysis (PLS-DA) was performed to investigate the discriminatory capacity of the metabolites across the clinical forms.

The robustness of the PLS-DA model was evaluated using cross-validation and a permutation test with 1000 random permutations to verify the model’s predictive capacity and reduce the risk of overfitting. Model quality was estimated by the *R*^2^ and *Q*^2^ parameters, and the model was considered statistically valid when the original values were superior to those of the permuted models and the permutation test demonstrated statistical significance (*p* < 0.05). Additionally, the relevance of each metabolite in discriminating among the groups was determined by Variable Importance in Projection (VIP) values. Heatmaps were employed to visualize the relative abundance of metabolites across the various clinical forms of leprosy.

In PLS-DA analyses, moderate explained variance values are frequently observed in clinical metabolomics studies involving heterogeneous biological systems, particularly in diseases of a spectral and multifactorial nature. Therefore, model interpretation should not rely exclusively on the percentage of explained variance; it is fundamental to consider validation metrics, such as the *R*^2^ and *Q*^2^ parameters, along with cross-validation and permutation tests, to assess statistical robustness and reduce the risk of overfitting [34].

## 3. Results

The epidemiological and clinical characteristics of the sample are presented in Table 1. The mean age of the participants was 45.27 years (SD ± 19.75), with a slight female predominance (52.7%). Detection of *Mycobacterium leprae* DNA via qPCR was positive in 96.4% of cases (53/55). The mean anti-PGL-I IgM EI was 2.86 (SD ± 3.37), while bacilloscopy was positive in 47.3% of cases (26/55), with a mean BI ≥ 2 (SD ± 2.52) (Table 1).

**Figure 2:**
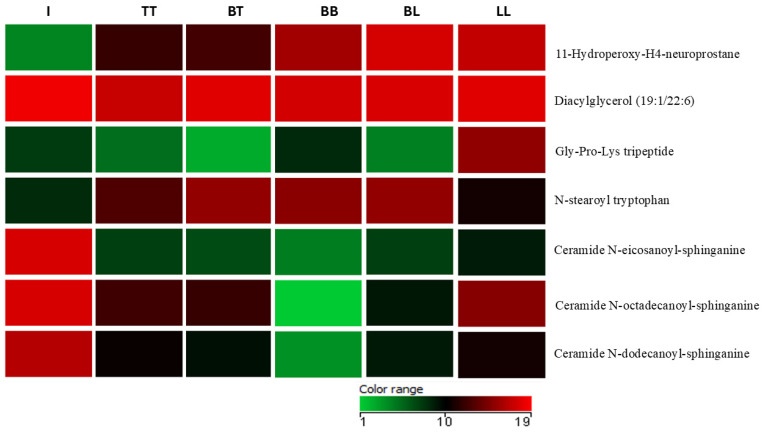
Heatmap displaying the mean normalized intensity of significant metabolites across the six clinical forms of the Ridley–Jopling spectrum: indeterminate (I), tuberculoid (TT), borderline tuberculoid (BT), mid-borderline (BB), borderline lepromatous (BL), and lepromatous (LL). The values represent group-level relative abundances obtained through the auto-scaling method (ranging from 1 to 19), with the color scheme indicating high (red) and low (green) relative metabolic intensities.

Six differentially expressed metabolites were identified across the clinical forms (Table 2 and Figure 2). Additionally, N-stearoyl tryptophan was included as a candidate metabolite based on its high Variable Importance in Projection (VIP) score in the multivariate model, despite showing no significant difference in group-wise univariate comparisons (*p* = 0.956).

The comparison across clinical forms using the Kruskal–Wallis test revealed statistically significant differences in the expression of most evaluated metabolites, including Gly-Pro-Lys tripeptide (H = 12.86; *p* = 0.025), N-eicosanoyl-sphinganine ceramide (H = 20.76; *p* = 0.001), N-octadecanoyl-sphinganine ceramide (H = 30.38; *p* < 0.001), N-dodecanoyl-sphinganine ceramide (H = 13.79; *p* = 0.017), 11-hydroperoxy-H4-neuroprostane (H = 13.50; *p* = 0.019), and DAG (H = 17.07; *p* = 0.004). In contrast, N-stearoyl-L-tryptophan showed no significant difference between the groups (H = 1.08; *p* = 0.956) (Table 2).

These findings indicate that specific metabolic alterations are associated with the clinical progression of leprosy, with a notable emphasis on complex lipids and metabolites derived from oxidative stress.

It should be noted that background controls, including tissue blanks (paraffin sections without tissue) and procedural extraction blanks, were not included in the original experimental design.

Heatmap analysis (Figure 2) revealed distinct patterns of relative metabolite abundance across the clinical forms of leprosy. A higher expression of lipid metabolites, particularly ceramides and diacylglycerol, was observed in multibacillary forms (BB, BL, and LL), whereas paucibacillary forms exhibited a lower relative intensity of these molecules. The Gly-Pro-Lys tripeptide demonstrated heterogeneous variation across the groups, with a more pronounced increase in the LL form. Overall, the heatmap underscores the presence of a metabolic gradient across the clinical spectrum, corroborating the heterogeneity observed in both univariate and multivariate analyses.

A consistent metabolic gradient pattern was observed across the Ridley–Jopling clinical spectrum, rather than isolated categorical differences. For instance, 11-hydroperoxy-H4-neuroprostane demonstrated a progressive increase in mean normalized intensity from the Indeterminate form (4.12) through the borderline forms to the Lepromatous form (18.84). This linear-like progression mirrored the rise in the bacillary index (BI) and anti-PGL-I levels, while inversely following the reduction in qPCR Ct values. These findings suggest that the metabolic signature of skin tissue reflects the ordered clinical–immunological continuum of the disease.

Similarly, clinical laboratory markers demonstrated behavior consistent with disease progression, with a progressive increase in the EI (0.90 to 7.22) and the biopsy-derived BI (0 to 5.77+), alongside the reduction in Ct values (37 to 19). These findings reinforce the internal data consistency and the robustness of the observed clinical immunological gradient.

Spearman’s correlation analysis revealed distinct associations between metabolites and markers of infection (Table 3). 11-hydroperoxy-H4-neuroprostane showed a weak but significant negative correlation with qPCR Ct values (*ρ* = −0.281; *p* = 0.038), indicating that higher expression is associated with a greater bacillary load.

On the other hand, the Gly-Pro-Lys tripeptide demonstrated weak to moderate significant correlations with all analyzed markers, including the BI (*ρ* = 0.338; *p* = 0.012), EI (*ρ* = 0.297; *p* = 0.028), and qPCR Ct values (*ρ* = −0.374; *p* = 0.005), standing out as the metabolite with the highest biological consistency among those evaluated. The remaining metabolites showed no significant correlations with clinical parameters, suggesting a role more closely related to the global metabolic phenotype than to the direct bacillary load.

To account for the continuous nature of the leprosy spectrum, a trend analysis revealed significant progressive changes in key metabolites. 11-hydroperoxy-H4-neuroprostane showed a statistically significant moderate positive correlation with clinical progression (*ρ* = 0.443; *p* = 0.001), indicating a gradual increase in expression from the indeterminate to the lepromatous pole. Similarly, the Gly-Pro-Lys tripeptide demonstrated a significant increasing trend across the spectrum (*ρ* = 0.323; *p* = 0.016). Conversely, Ceramide N-eicosanoyl-sphinganine exhibited a significant negative correlation (*ρ* = −0.275; *p* = 0.042), suggesting a progressive reduction in expression in advanced multibacillary forms.

**Figure 3:**
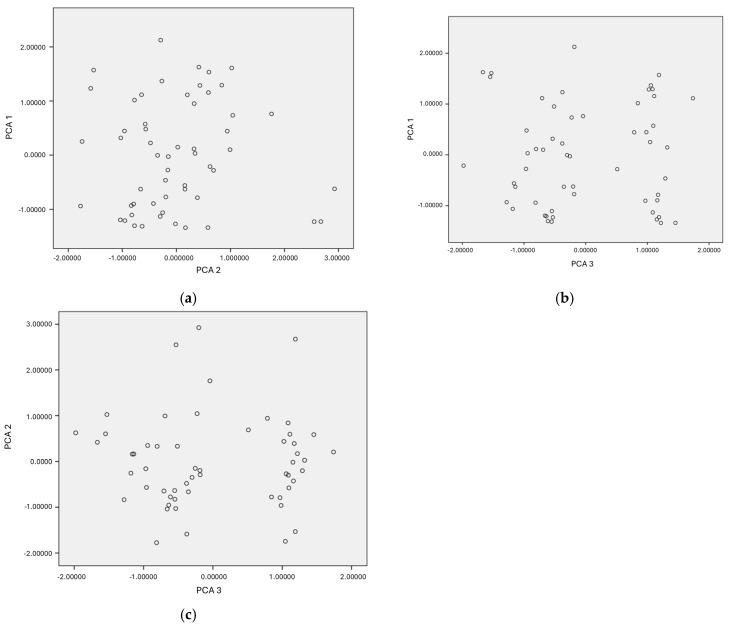
Principal Component Analysis (PCA) of metabolomic profiles from FFPE skin biopsies across the clinical spectrum of leprosy. (**a**) The PC1 versus PC2 score plot, which accounts for the largest proportion of total data variance, shows no clear separation between clinical forms, highlighting significant overlap between groups and high metabolic heterogeneity. (**b**) The PC1 versus PC3 score plot shows a partial improvement in group distribution along the clinical spectrum, although considerable overlap among samples remains. (**c**) The PC2 versus PC3 analysis reveals a slight trend toward spatial organization among some clinical forms, suggesting an additional contribution of the third principal component to metabolic differentiation. These findings reinforce the continuous and heterogeneous nature of the immunometabolic response in leprosy across the Ridley–Jopling spectrum. The first three principal components explain 68.1% of the total data variance.

Principal component analysis (PCA) demonstrated data adequacy for dimensional reduction (KMO = 0.670; Bartlett’s test *p* < 0.001), with the first three components explaining 68.1% of the total variance. The projection of samples onto different factorial planes revealed a heterogeneous distribution, with no well-defined clusters forming among the clinical forms. Although the inclusion of the third component revealed a slight additional organization of the data, considerable overlap was observed between the groups, indicating an absence of clear discrimination in an unsupervised approach (Figure 3).

Supervised partial least squares-discriminant analysis (PLS-DA) demonstrated partial discrimination across the different clinical forms of leprosy, evidencing a metabolic clustering tendency along the clinical spectrum. The model presented moderate explanatory capacity, with an *R*^2^ of approximately 0.46, and discrete predictive capacity, with a *Q*^2^ of approximately 0.26.

**Figure 4:**
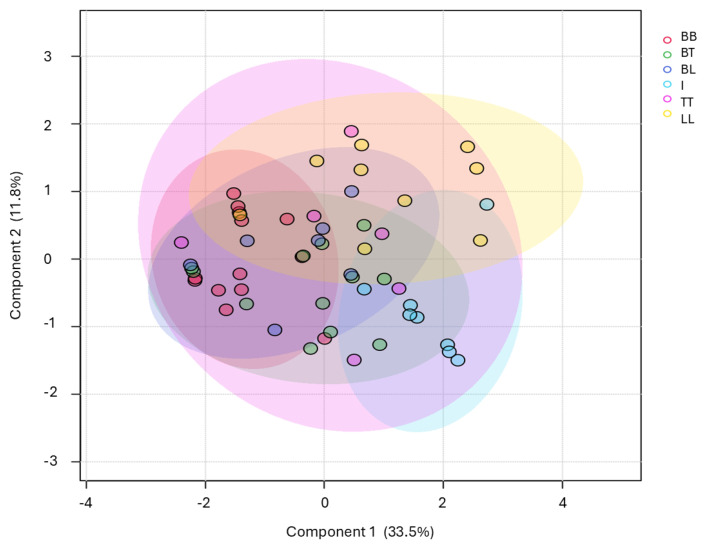
Partial Least Squares-Discriminant Analysis (PLS-DA) score plot of metabolomic profiles from FFPE skin biopsies across the clinical spectrum of leprosy. Principal components PC1 and PC2 account for 33.5% and 11.8% of the total data variance, respectively. A heterogeneous distribution of samples is observed across clinical groups, showing a partial separation tendency along the disease’s immunological spectrum. The indeterminate (I) and tuberculoid (TT) forms, characterized by robust cell-mediated immunity and low bacillary load, clustered predominantly in the negative and intermediate regions of PC1. The borderline forms (BT, BB, and BL) demonstrated higher spatial dispersion, reflecting the immunological instability and metabolic heterogeneity typical of these intermediate states. The lepromatous (LL) form, associated with high bacillary load and predominant humoral immunity, was primarily concentrated in the lower regions of PC2, suggesting a distinct metabolic signature. The ellipses represent the intragroup dispersion and variability for each clinical form.

Multivariate PLS-DA demonstrated partial separation between the clinical forms, showing a tendency toward sample clustering despite significant overlap between the groups. The first two components of the PLS-DA model explained 45.3% of the data variability, indicating an adequate representation of the metabolic structure (Figure 4). Partial overlap was observed between the clinical groups, especially among the borderline forms; however, a trend toward progressive distribution along the clinical spectrum was evident, consistent with the continuous nature of the Ridley–Jopling classification.

Model cross-validation demonstrated improved performance with two principal components compared to a single component, evidencing an increase in the explanatory and predictive capacity of the multivariate model.

The statistical robustness of the supervised analysis was confirmed by a permutation test with 1000 random permutations, demonstrating statistical significance (*p* = 0.023). This indicates that the observed discrimination among the clinical groups was unlikely to have occurred by chance. Although only 23 of the 1000 permuted models presented similar or superior performance compared to the original, the model displayed limited predictive capacity (Q^2^ ≈ 0.26), consistent with the overlapping immunometabolic nature of the leprosy spectrum.

**Figure 5:**
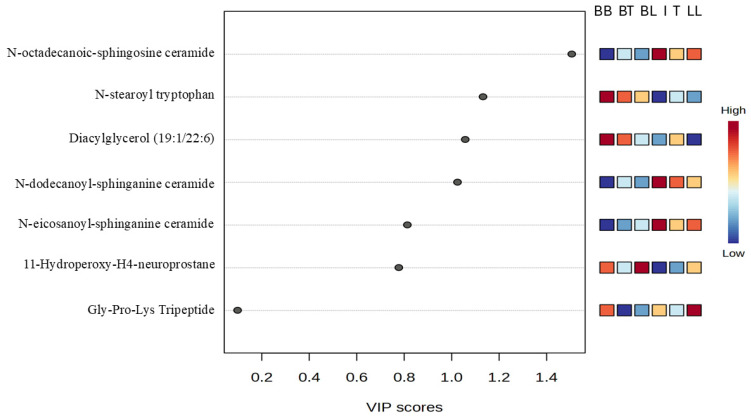
Contribution of metabolites to the discrimination among the clinical forms of leprosy based on Variable Importance in Projection values.

Variable Importance in Projection (VIP) analysis within the PLS-DA model revealed differences in the contribution of metabolites to the discrimination among the clinical forms of leprosy (Figure 5). The highest VIP values were observed for N-octadecanoyl-sphinganine ceramide (VIP ≈ 1.5), followed by N-stearoyl-L-tryptophan (VIP ≈ 1.1) and diacylglycerol (VIP ≈ 1.0), indicating the greater relevance of these metabolites in the multivariate model. N-dodecanoyl-sphinganine ceramide presented a borderline value (VIP ≈ 1.0), while N-eicosanoyl-sphinganine (VIP ≈ 0.8) and 11-hydroperoxy-H4-neuroprostane (VIP ≈ 0.7) demonstrated moderate contributions. In contrast, the Gly-Pro-Lys tripeptide presented a low VIP value (≈0.2), indicating a minor role in the global discrimination between the groups.

A critical limitation of this study is the absence of a healthy (non-leprosy) control group or a cohort with other inflammatory dermatological conditions. While the study’s primary goal was to characterize metabolic variability across the Ridley–Jopling spectrum, the lack of such controls precludes a definitive assessment of whether the identified signatures are unique to leprosy or reflect general skin inflammation. However, the consistent correlation between metabolites like the Gly-Pro-Lys tripeptide and 11-hydroperoxy-H4-neuroprostane with specific leprosy markers (BI, qPCR, and anti-PGL-I) provides evidence of their direct relationship with the disease’s pathophysiology. In the context of the biomarker hierarchy, these metabolic features should be interpreted as discovery-to-candidate signals that provide mechanistic insight into host–pathogen immunometabolic interactions rather than clinically validated tools. As metabolomics in leprosy remains largely exploratory, these findings represent an innovative strategy for utilizing archived FFPE samples for translational research, pending future validation in larger, longitudinal cohorts that include diverse dermatological control groups.

Furthermore, a methodological limitation of this study is the omission of paraffin-only tissue blanks and extraction blanks, which are typically used to subtract background signals from embedding media and fixation chemicals. Nevertheless, the biological relevance of our findings is supported by the robust correlations between the identified metabolites and established quantitative markers of leprosy (BI, qPCR, and anti-PGL-I). The consistency of these results across the Ridley–Jopling spectrum suggests that the identified signatures are biologically derived from the skin tissue and reflect host–pathogen immunometabolic interactions.

The model cross-validation failed to identify features with robust statistical significance, indicating limited predictive power. While the permutation test confirmed that the PLS-DA model’s discrimination was statistically significant (*p* = 0.023), the model cross-validation demonstrated limited predictive power (*Q*^2^ ≈ 0.26). This does not imply that the discrimination occurred by chance, but rather that the global metabolomic profile exhibits significant overlap between adjacent clinical forms. This pattern reflects the continuous pathophysiology of leprosy, where transitions between the Ridley–Jopling categories are characterized by metabolic gradients rather than isolated biological states. Consequently, while these signatures provide valuable mechanistic insights, they require further validation in larger cohorts before they can be considered robust predictive tools. Study limitations include the relatively small number of metabolites included in the analysis and the absence of a healthy (non-leprosy) control group. While the primary purpose of this study was to characterize the metabolic variability across the Ridley–Jopling clinical spectrum, the lack of a control group precludes an absolute assessment of whether the identified metabolites are unique to leprosy or reflect general processes of skin inflammation or other infections. Nevertheless, the consistent correlation between metabolites like the Gly-Pro-Lys tripeptide and specific leprosy markers (BI, qPCR, and anti-PGL-I) provides evidence of their relationship with the disease’s pathophysiology. Future longitudinal research with broader metabolomic coverage and the inclusion of both healthy and other dermatological control groups will be essential to further validate these findings and determine their diagnostic specificity.

## 4. Discussion

The findings of this study demonstrate that the metabolomic profile associated with the various clinical forms of leprosy exhibits significant alterations, although characterized by high heterogeneity and overlap between groups, reflecting the continuous nature of the disease’s clinical spectrum.

Univariate analysis revealed significant differences in the expression of most evaluated metabolites, particularly ceramides and lipid derivatives, suggesting a central role for lipid metabolism in the pathophysiology of leprosy. These results are consistent with current knowledge indicating that *Mycobacterium leprae* modulates host lipid metabolism, favoring the formation of lipid-rich intracellular niches and contributing to bacterial persistence.

Among the metabolites analyzed, the Gly-Pro-Lys tripeptide stood out for its consistent correlation with all markers of bacillary load and humoral response, including the bacillary index (BI), anti-PGL-I ELISA index (EI), and qPCR Ct values. This pattern suggests that Gly-Pro-Lys may be related to systemic processes associated with infection progression, possibly reflecting alterations in protein degradation, inflammatory response, or tissue remodeling [36,37,38].

Recent metabolomics and lipidomics studies in leprosy consistently indicate that altered metabolic pathways primarily reflect host–pathogen immunometabolic interactions rather than validated clinical biomarkers. Within the biomarker hierarchy framework, metabolic features identified in exploratory studies—including those reported here—remain at the discovery-to-candidate stage and require further independent validation before clinical translation.

In this context, the associations observed for Gly-Pro-Lys and 11-hydroperoxy-H4-neuroprostane are more appropriately interpreted as indicators of host immunometabolic responses associated with bacillary burden and disease progression. It is important to emphasize that these correlations, while statistically significant, were of moderate magnitude and should not be interpreted as evidence of causality. As highlighted in recent reviews, although omics approaches have generated valuable insights into disease-associated metabolic pathways, the heterogeneity of methodologies has hindered clinical translation. Therefore, the findings in the present study should be regarded as putative candidate signatures that contribute to the understanding of lipid remodeling and oxidative stress in the pathophysiology of leprosy, rather than as standalone diagnostic or prognostic tools.

On the other hand, 11-hydroperoxy-H4-neuroprostane showed a significant correlation only with qPCR Ct values, indicating a more specific association with bacillary load, potentially related to oxidative stress and lipid peroxidation processes induced by the infection [39,40,41]. Unsupervised multivariate analysis (PCA) did not reveal clear separation between clinical forms, even after the inclusion of the third principal component, indicating that the global metabolomic profile is insufficient to completely discriminate the groups. This finding underscores the biological complexity of leprosy and aligns with the Ridley–Jopling classification, which describes the disease as a continuous spectrum where intermediate forms exhibit overlapping immunological and clinical features [2,38,39,40]. Collectively, these results indicate that while the global profile shows overlap, specific metabolic alterations involving complex lipids and metabolites derived from oxidative stress are associated with the clinical progression of leprosy.

Complementarily, supervised partial least squares-discriminant analysis (PLS-DA) demonstrated partial discrimination among the clinical forms of leprosy, evidencing a metabolic clustering tendency along the Ridley–Jopling spectrum. The model presented moderate explanatory capacity (*R*^2^ ≈ 0.46) and discrete predictive capacity (*Q*^2^ ≈ 0.26), indicating that part of the metabolic variability between the groups was captured by the multivariate model. Despite the moderate predictive capacity, validation by permutation test demonstrated statistical significance (*p* = 0.023; 1000 permutations), confirming that the observed discrimination did not occur by chance and reducing the probability of overlap. These results suggest the presence of biologically relevant metabolomic signatures associated with the different clinical forms, even though metabolic overlap exists between the groups.

VIP analysis revealed that metabolites related to lipid metabolism were the primary factors responsible for the discrimination among clinical forms. N-octadecanoyl- sphinganine ceramide presented the highest VIP value (>1.5), identifying it as the metabolite with the greatest contribution to multivariate separation among the clinical groups. Subsequently, N-stearoyl tryptophan and N-dodecanoyl-sphinganine ceramide stood out with values near or above 1.0, indicating a relevant contribution to the discriminant model. In contrast, 11-hydroperoxy-H4-neuroprostane and the Gly-Pro-Lys tripeptide presented lower VIP values (≈0.7 and ≈0.2, respectively), despite their biological association with increased bacillary load and oxidative stress in multibacillary forms. These findings demonstrate that biologically relevant metabolites do not always correspond to the primary statistical discriminators in multivariate analyses.

A notable finding in this study is the discrepancy between the robust biological consistency of the Gly-Pro-Lys tripeptide in univariate analyses and its limited contribution to the supervised multivariate model (VIP ≈ 0.2). While Gly-Pro-Lys showed the most significant and consistent correlations with the bacillary index, anti-PGL-I serology, and qPCR Ct values, its low VIP score indicates it is not a primary driver of overall group separation. This distinction suggests that Gly-Pro-Lys acts as a functional readout of disease activity—potentially reflecting tissue remodeling and protein degradation processes associated with infection—that is distributed across the continuous Ridley–Jopling spectrum rather than being unique to a specific clinical form. Within the biomarker hierarchy framework, this tripeptide represents a discovery-to-candidate pathophysiological signature. While its limited multivariate contribution reflects the inherent immunometabolic overlap between adjacent leprosy forms, its strong correlation with bacillary load identifies it as a biologically relevant marker for investigating host response dynamics.

The absence of robust separation observed in the multivariate PCA and PLS-DA analyses likely reflects the inherent biological complexity and the continuous nature of the clinical spectrum of leprosy. Unlike diseases with clearly distinct metabolic phenotypes, the various clinical forms of leprosy share common immunological, inflammatory, and metabolic mechanisms, resulting in considerable overlap between metabolomic profiles. This pattern suggests an immunometabolic continuity along the disease spectrum, with gradual transitions between clinical forms rather than biologically isolated groups. Additionally, the shared metabolic pathways related to lipid metabolism, oxidative response, and host–pathogen interaction likely contribute to the heterogeneity observed across the groups. Thus, the partial discrimination identified by the multivariate analyses appears to reflect the continuous pathophysiology of leprosy rather than a methodological limitation of the study.

Although the present study represents one of the larger metabolomic investigations in leprosy using FFPE tissue, the sample size (n = 55) remains a limiting factor for multivariate modeling and biomarker discovery. Metabolomic data are inherently high-dimensional, and smaller cohorts may increase the risk of overfitting, reduce statistical power, and limit the generalizability of identified metabolic signatures. Therefore, while the observed associations—particularly those involving lipid-derived metabolites and oxidative stress markers—are biologically plausible and consistent with disease mechanisms, they should be interpreted as exploratory and hypothesis-generating rather than definitive biomarkers. Larger, independent cohorts will be necessary to validate the robustness and reproducibility of these findings across different populations and analytical platforms.

It is important to emphasize that several of the observed associations, particularly those derived from univariate correlations, were of moderate magnitude and should not be interpreted as evidence of causality. In metabolomic studies, correlation patterns may be influenced by biological variability, analytical noise, and multiple testing effects. Therefore, metabolites such as Gly-Pro-Lys and 11-hydroperoxy-H4-neuroprostane should be considered putative biomarkers associated with disease activity, while N-stearoyl tryptophan represents a multivariate candidate signature that contributed to model discrimination despite high individual variability across clinical forms. The multivariate results further support this caution, as biologically relevant metabolites did not always coincide with the strongest statistical discriminators (VIP scores), highlighting the distinction between biological relevance and statistical contribution in high-dimensional datasets.

The partial discrimination observed in multivariate analyses, combined with the progressive increase in markers like 11-hydroperoxy-H4-neuroprostane, reinforces the continuous pathophysiology of leprosy. Rather than representing biologically isolated groups, the clinical forms share overlapping immunometabolic mechanisms that transition gradually along the Ridley–Jopling spectrum. The trend analysis reinforces the biological interpretation that metabolomic alterations in skin tissue accompany the progressive immunometabolic shifts in the disease. The gradual increase in 11-hydroperoxy-H4-neuroprostane and Gly-Pro-Lys, alongside the reduction in specific ceramide species, suggests that these signatures reflect continuous functional readouts of disease activity rather than differences restricted to isolated clinical groups. These findings strengthen the evidence that metabolic remodeling is intrinsically linked to the increasing bacillary burden and the decline of cell-mediated immunity toward the lepromatous pole. By treating the Ridley–Jopling classification as an ordered clinical–immunological continuum, this analysis provides a more informative capture of the metabolic transitions occurring across the disease spectrum, moving beyond simple group-wise comparisons to highlight the continuous nature of leprosy’s pathophysiology.

While the biological interpretation of the identified metabolites—such as Gly-Pro-Lys and 11-hydroperoxy-H4-neuroprostane—provides mechanistic insight into host–pathogen interactions, their translation into clinically actionable tools requires a structured hierarchical framework. Currently, these features should be interpreted as pathophysiological signatures at the discovery-to-candidate stage rather than validated diagnostic biomarkers.

Nevertheless, the potential clinical utility of these signatures lies in their ability to serve as functional readouts of disease activity and bacillary burden. For instance, the progressive increase in oxidative stress markers across the Ridley–Jopling spectrum suggests that metabolomics could supplement current classification methods (BI, anti-PGL-I, and qPCR) by providing a more granular estimation of metabolic dysfunction in highly infected tissues. Furthermore, since previous studies indicate that metabolic alterations often normalize following the completion of multidrug therapy (MDT), these candidates may eventually prove useful for monitoring treatment response and detecting early signs of treatment failure.

Regarding immunological complications, the identification of metabolites associated with acute inflammatory states—as seen in urinary metabolomics studies of type 1 reactions—suggests that skin-derived lipid signatures could potentially serve as early indicators of leprosy reactions or neurological damage before clinical onset. However, we emphasize that these applications remain hypothetical. Future research must prioritize longitudinal cohorts to determine if these candidate signatures can accurately predict long-term outcomes and reactive episodes, bridging the gap between molecular discovery and clinical implementation.

Study limitations include the relatively small number of metabolites included in the analysis, which may restrict the discriminative capacity of multivariate models. Additionally, the cross-sectional nature of the study precludes the evaluation of metabolic changes over time. The absence of a non-leprosy control group limits the assessment of global metabolic signatures associated with the disease, restricting the analyses to comparisons among the various clinical forms of the leprosy spectrum. A critical concern in the use of archived tissue is the potential for metabolite degradation during long-term storage. In this study, FFPE blocks were stored for 3 to 8 years (2015–2020) prior to extraction. While chemical alterations like oxidation or cross-linking may occur, the literature suggests that detectable metabolic information can be retained for decades [42,43,44]. The robust correlations found between key metabolites and quantitative clinical markers (BI, qPCR) strongly suggest that biologically relevant signatures were preserved despite the archival period. These findings reinforce the viability of using FFPE biopsies for retrospective metabolomic investigations, confirming that even years after collection, these samples can yield reliable annotations that discriminate the clinical forms of leprosy. Thus, the partial discrimination observed in our multivariate models appears to reflect the inherent biological complexity of the disease’s continuous spectrum rather than a loss of data integrity due to archival aging.

Another relevant limitation is the potential influence of confounding clinical and demographic variables, such as age, sex, comorbidities, nutritional status, and treatment history, which were not fully controlled in the present analysis. These factors are known to influence systemic lipid metabolism and inflammatory pathways, potentially affecting the observed metabolomic profiles. To further mitigate these effects, the study implemented strict exclusion criteria for major infectious comorbidities, including tuberculosis, HIV/AIDS, and viral hepatitis. However, in a retrospective, cross-sectional design, residual confounding from factors such as nutritional status, environmental influences, and individual medical histories cannot be completely excluded. Although the grouping by clinical form of leprosy likely captures the major biological gradient of disease severity, residual confounding cannot be excluded. Future studies incorporating multivariable regression models or stratified analyses will be necessary to disentangle disease-specific metabolic signatures from host-related variability.

## 5. Conclusions

This study demonstrated that the metabolomic profile associated with the various clinical forms of leprosy exhibits significant alterations, particularly involving lipid metabolites and oxidative stress derivatives. Although univariate analyses revealed consistent differences between the groups, the multivariate approach indicated limited global discrimination capacity, reflecting the continuous and heterogeneous nature of the disease’s clinical spectrum. Among the analyzed metabolites, the Gly-Pro-Lys tripeptide and 11-hydroperoxy-H4-neuroprostane stood out as markers associated with bacillary load and bacterial DNA levels. In contrast, ceramides were more relevant for discriminating between clinical forms in the multivariate context, reinforcing the role of lipid metabolism in the pathophysiology of leprosy.

Despite its contributions, this study has limitations, including a relatively small sample size (*n* = 55), a cross-sectional design that precludes longitudinal monitoring, and the absence of a healthy control group to confirm diagnostic specificity against other inflammatory skin conditions. Notably, however, this study utilized FFPE tissue samples, demonstrating the viability of this approach in routinely available material. This strategy significantly expands the potential for retrospective research using archived biological collections. Future research should focus on longitudinal designs with broader metabolomic coverage and the inclusion of diverse control groups to validate these candidate biomarkers. Furthermore, the application of FFPE-based metabolomics should be explored in other infectious and neglected tropical diseases to leverage existing histopathological archives for translational discovery. Collectively, these results highlight the potential of individual metabolites as complementary tools in investigating leprosy pathophysiology and monitoring disease progression.

## Figures and Tables

**Table 1:** Epidemiological, clinical, and laboratory variables of the study participants (n = 55).

Variable	n	%
**Sex**		
Male	26	47.0
Female	29	53.0
**Age**		
≤15 years	8	14.5
>15 years	47	85.5
**Clinical Form**		
Indeterminate	8	14.5
Tuberculoid	6	11.0
Borderline tuberculoid	12	21.8
Mid-borderline	13	23.6
Borderline lepromatous	7	12.7
Lepromatous	9	16.4
Total	55	100
**Biopsy-Derived Bacillary Index**		
0	30	54.5
1+	3	5.4
2+	2	3.6
3+	0	0
4+	6	11.0
5+	4	7.3
6+	10	18.2
**Biopsy qPCR**		
Negative	2	3.6
Positive	53	96.4
**Anti-PGL-I IgM ELISA**		
<1.0 (Negative)	19	34.5
≥1.0 (Positive)	36	65.5

**Table 2:** Expression intensity of differentially expressed and candidate metabolites, ELISA Index (EI), biopsy-derived bacillary index (BI), and qPCR cycle threshold (Ct) values according to the clinical forms of the Ridley–Jopling spectrum.

Metabolites ^1^		Clinical Forms						
		I (n = 8)	TT (n = 6)	BT (n = 12)	BB (n = 13)	BL (n = 7)	LL (n = 9)	Kruskal–Wallis (*p*)
11-Hydroperoxy-H4-neuroprostane	$\bar{x}$	4.12	11.91	12.35	15.72	17.52	18.84	*p* = 0.019
	±SD	7.71	9.25	9.18	7.12	1.15	6.46	
Diacylglycerol (19:1/22:6)	$\bar{x}$	18.46	17.02	17.93	17.39	17.65	17.93	*p* = 0.04
	±SD	0.34	0.60	1.24	0.45	0.55	0.85	
Gly-Pro-Lys tripeptide	$\bar{x}$	7.39	5.13	2.52	8.25	4.35	15.08	*p* = 0.025
	±SD	7.90	7.95	5.88	7.95	7.43	0.13	
N-stearoyl tryptophan ^2^	$\bar{x}$	8.16	12.73	15.14	14.85	15.20	10.65	*p* = 0.956
	±SD	8.72	6.29	1.16	0.54	0.59	8.04	
N-eicosanoyl-sphinganine ceramide	$\bar{x}$	17.55	7.19	6.71	4.50	7.16	8.81	*p* = 0.001
	±SD	1.33	7.88	8.41	7.03	8.93	8.39	
N-octadecanoyl-sphinganine ceramide	$\bar{x}$	17.60	12.20	11.90	1.10	8.99	14.72	*p* = 0.000
	±SD	1.19	5.99	7.25	3.97	8.49	5.86	
N-dodecanoyl-sphinganine ceramide	$\bar{x}$	16.34	10.31	9.33	3.60	8.83	10.68	*p* = 0.017
	±SD	0.81	8.01	8.29	6.85	8.28	8.05	
	**Clinical and Laboratory Variables**							
Mean ELISA Index ^3^	$\bar{x}$	0.90	0.74	1.62	1.98	5.13	7.22	*p* = 0.000
	±SD	0.55	0.86	1.30	1.75	4.27	4.34	
Mean Biopsy Bacillary Index ^4^	$\bar{x}$	0	0	0.16	2.23	4.00	5.77	*p* = 0.000
	±SD	0	0	0.38	2.16	2.23	0.44	
Mean qPCR Ct value ^5^	$\bar{x}$	37	35	33	26	25	19	*p* = 0.000
	±SD	1.30	14.55	10.04	5.73	6.70	3.04	

^1^ Mean normalized intensity of metabolites expressed as a relative value obtained by the auto-scaling method (0–19), where zero (0) indicates an unidentified metabolite and 19 indicates a highly expressed metabolite. ^2^ N-stearoyl tryptophan is considered a candidate metabolite based on its multivariate contribution (VIP > 1.0) rather than univariate significance. ^3^ Mean anti-PGL-I antibody titers expressed as ELISA Index (EI), calculated by the formula: EI = ODsample/ODcutoff, where values ≥ 1.0 are considered positive. ^4^ Mean Biopsy Bacillary Index (BI), ranging from 0 to 6+, where 0 indicates absence of bacilli and 6 indicates high bacillary load (>1000 bacilli/field). ^5^ Ct = cycle threshold: Ct = 0: negative; Ct < 40: positive.

**Table 3:** Spearman’s correlation (*ρ*) between individual metabolite expression and markers of bacillary load and humoral response (*p* < 0.05).

Metabolite	Biopsy Bacillary Index	Anti-PGL-I ELISA Index	qPCR Cycle Threshold
11-Hydroperoxy-H4-neuroprostane	*ρ* = 0.224 *p* = 0.100	*ρ* = 0.193 *p* = 0.158	*ρ* = −0.281 *p* = 0.038
Diacylglycerol (19:1/22:6)	*ρ* = 0.037 *p* = 0.786	*ρ* = 0.184 *p* = 0.179	*ρ* = 0.028 *p* = 0.839
Gly-Pro-Lys tripeptide	*ρ* = 0.338 *p* = 0.012	*ρ* = 0.297 *p* = 0.028	*ρ* = −0.374 *p* = 0.005
N-stearoyl tryptophan	*ρ* = 0.051 *p* = 0.711	*ρ* = 0.093 *p* = 0.501	*ρ* = −0.221 *p* = 0.104
N-eicosanoyl-sphinganine ceramide	*ρ* = −0.100 *p* = 0.468	*ρ* = −0.124 *p* = 0.368	*ρ* = 0.156 *p* = 0.256
N-octadecanoyl-sphinganine ceramide	*ρ* = −0.079 *p* = 0.564	*ρ* = 0.016 *p* = 0.909	*ρ* = 0.135 *p* = 0.327
N-dodecanoyl-sphinganine ceramide	*ρ* = −0.093 *p* = 0.500	*ρ* = 0.013 *p* = 0.923	*ρ* = −0.032 *p* = 0.817

## Data Availability

The data presented in this study are available in Supplementary Material Table S1.

## Supplementary Materials

The following supporting information can be downloaded at https://www.mdpi.com/article/10.3390/microorganisms14071567/s1. Table S1: Individual clinical and laboratory data of the study participants.

## Abbreviations

The following abbreviations are used in this manuscript:

ANOVA	Analysis of Variance
BB	Mid-borderline leprosy
BI	Bacillary Index
BL	Borderline lepromatous leprosy
BT	Borderline tuberculoid leprosy
Ct	Cycle threshold (qPCR)
DAG	Diacylglycerol
EI	ELISA Index
ELISA	Enzyme-Linked Immunosorbent Assay
FDR	False Discovery Rate
FFPE	Formalin-Fixed Paraffin-Embedded
I	Indeterminate leprosy
LC-MS	Liquid Chromatography–Mass Spectrometry
LL	Lepromatous leprosy
MB	Multibacillary
PB	Paucibacillary
PCA	Principal Component Analysis
PLG-I	Phenolic Glycolipid-I
PLS-DA	Partial Least Squares-Discriminant Analysis
qPCR	quantitative Polymerase Chain Reaction
TT	Tuberculoid leprosy
VIP	Variable Importance in Projection
