# Supplementary material for: Mass Spectrometry-Based Metabolomics in Formalin-Fixed Paraffin-Embedded Skin Biopsies Identifies Potential Candidate Biomarkers for Leprosy Progression Across the Ridley–Jopling Clinical Spectrum
Source: Microorganisms. 2026 Jul 17;14(7):1567. doi: 10.3390/microorganisms14071567 (PMC13414152; doi:10.3390/microorganisms14071567)
Supplement: Supplementary file 1 [file microorganisms-14-01567-s001.zip › microorganisms-4364542-supplementary.pdf]

Table S1- Material Suplementar

CONTROLE	FORMA CLÍNICA	SEXO	IDADE	qPCR(cycle threshold)	ELISA	INDICE BACILOSCOPICO DA BIOPSIA
**51**	**I**	F	59	36	0.58	0
**52**	**I**	F	26	35	0.45	0
**53**	**I**	M	13	34	0.51	0
**54**	**I**	F	9	34	1.72	0
**55**	**I**	F	21	36	0.63	0
**57**	**I**	F	49	36	1.36	0
**58**	**I**	F	48	38	0.39	0
**59**	**I**	F	12	36	1.59	0
**1**	T	F	11	36	0.46	0
**2**	T	M	18	34	0.2	0
**3**	T	F	41	35	0.32	0
**4**	T	F	7	32	0.43	0
**5**	T	F	18	39	0.54	0
**6**	T	M	43	0	0.44	0
**7**	T	M	39	0	0.71	0
**9**	T	F	5	0	0.38	0
**10**	**T**	F	24	0	2.5	0
**8**	DT	F	50	0	0.42	0
**11**	**DT**	M	60	36	1.6	1
**12**	**DT**	M	42	36	1.02	0
**13**	**DT**	M	72	35	0.59	0
**14**	**DT**	M	46	36	0.74	0
**15**	**DT**	F	40	31	1.58	0
**16**	**DT**	F	7	33	2.38	0
**17**	**DT**	F	51	36	0.49	0
**19**	**DT**	F	67	30	3.8	1
**20**	**DT**	M	46	35	0.66	0
**32**	DT	M	64	33	0.38	0
**56**	**DT**	F	51	0	1.88	0
**60**	**DT**	F	32	34	4.35	0
**18**	**DD**	F	45	36	0.07	0
**21**	DD	M	62	21	6.2	4
**22**	DD	M	53	25	2.43	4
**23**	DD	M	65	25	1.23	4
**24**	DD	M	53	30	1.07	2
**25**	DD	F	49	30	0.1	0
**26**	DD	M	70	35	2.03	0
**27**	DD	M	63	33	0.3	1
**28**	DD	F	60	32	1.74	0
**29**	DD	F	71	23	3.1	4

**30**	DD	F	63	36	1.36	0
**36**	**DD**	M	76	24	1.76	6
**39**	**DD**	M	53	20	4.37	4
**31**	DV	M	51	36	5.47	2
**33**	DV	F	59	30	0.67	0
**34**	**DV**	M	45	24	2.46	5
**35**	**DV**	M	66	15	3.99	6
**37**	**DV**	M	13	24	12.39	6
**38**	**DV**	F	30	29	9.22	4
**40**	**DV**	M	62	22	1.73	5
**41**	VV	F	91	22	0.58	6
**42**	VV	M	48	19	5.36	5
**43**	VV	M	57	18	14.73	6
**44**	VV	F	57	17	8.7	6
**45**	VV	F	42	23	5.64	6
**46**	VV	M	71	14	10.93	6
**47**	VV	M	15	16	3.17	6
**48**	VV	F	43	20	2.09	6
**49**	VV	M	52	22	11.03	6
**50**	VV	F	62	22	3.34	5
